# DYADIC APPRAISALS OF FAMILY DECISIONS AND HEALTH TASKS IN MIDDLE-AGED AND OLDER COUPLES

**DOI:** 10.1093/geroni/igac059.2335

**Published:** 2022-12-20

**Authors:** Karen Lyons, Sae Hwang Han, Kyungmin Kim, Lyndsey Miller

**Affiliations:** Boston College, Chestnut Hill, Massachusetts, United States; University of Texas at Austin, Austin, Texas, United States; Seoul National University, Seoul, Seoul-t'ukpyolsi, Republic of Korea; Oregon Health & Science University, Portland, Oregon, United States

## Abstract

Dyadic theories, including the Theory of Dyadic Illness Management, emphasize the important protective roles of shared appraisals and collaborative tasks within the couple for optimizing health and well-being. Within illness contexts, research has also focused on shared decision-making and collaborative illness management within couples. However, less is known about how concordant middle-aged and older couples are regarding how they make decisions (e.g., one partner has final say, both have equal say) and how they complete health-related tasks (e.g., one partner does it, they do it together), factors associated with being more concordant as a couple, and implications of concordance for health and well-being of both members. Data from 2,761 couples (Mage = 64.94 for wives and 68.04 for husbands) from the Health and Retirement Study (2014/16–2016/18) were examined. The majority of couples were congruent regarding who makes family decisions (69.7%) and who completes medical forms (64.4%) within the couple; further, 62% agreed they make family decisions collaboratively versus 25.5% completing medical forms collaboratively. Greater concordance in these appraisals was significantly associated with greater marital support and length of marriage. While concordance in appraisals was not significantly associated with depressive symptoms 2 years later, the link between congruence in making major family decisions and self-rated health differed by age and gender— suggesting that the intersection of age and gender may shape how decisions within couples lead to potential health benefits.

